# A Pilot Study to Develop the Rapid Estimate of Adult Literacy in Audiology

**DOI:** 10.3928/24748307-20220418-01

**Published:** 2022-04

**Authors:** Hua Ou

## Abstract

**Background:**

Health literacy describes an individuals' ability to maximize their potential in health care, including one's ability to understand information needed to make informed health decisions. A variety of general and condition-specific health literacy assessment tools have been created to help health professionals assess patients' health literacy skills and tailor the need for health care communication or education; however, there are no such tools available for the audiology field.

**Objective:**

The purpose of the study was to develop an objective reading recognition audiology-related health literacy assessment tool, the Rapid Estimate of Adult Literacy in Audiology (REALA).

**Methods:**

This was a cross-sectional study (*N* = 200). The initial version of the REALA contained 99 words specifically related to audiology. The final version, revised to have improved clinical utility, contained a total of 48 words that were selected based on item difficulty, item discrimination score, and point-biserial index using classical item analysis.

**Key Results:**

The total pass rate for the final version of the 48-word REALA was 0.72 (standard deviation = 0.45) and the Cronbach coefficient alpha was 0.93. Once the comprehension component is added to the tool, the REALA can be a valuable health literacy assessment tool that health professionals use to evaluate patients' audiology-related health literacy.

**Conclusion:**

Once the comprehension component is added to the tool, the REALA can be a valuable health literacy assessment tool that health professionals use to evaluate patients' audiology-related health literacy. **[*HLRP: Health Literacy Research and Practice*. 2022;6(2):e88–e95.]**

**Plain Language Summary:**

A health literacy assessment tool, the REALA, was developed in the study. The final version of REALA contained 48 words relative to hearing healthcare. The results suggested that REALA can help health professionals assess patients' hearing related health literacy and tailor the need for hearing health care communication or education.

Health literacy is defined as “the degree to which individuals have the capacity to obtain, process, and understand basic health information and services needed to make appropriate health decisions” (U.S. Department of Health and Human Services, Office of Disease Prevention and Health Promotion, 2000). A review of health literacy interventions and outcomes revealed that differences in health literacy were associated with increased hospitalizations and emergency department visits, poorer adherence to recommendations regarding medications, and lower use of screening and preventative services such as mammography or getting the influenza vaccination (Berkman et al., 2011). Therefore, increasing the use of evidence-based health literacy practices is imperative to improve health care quality.

There are a variety of tools and approaches used to assess a person's health literacy. For example, one of the most widely used tools, the Rapid Estimate of Adult Literacy in Medicine (REALM), is a reading recognition test that can identify low levels of health literacy among patients (Davis et al., 1993). Reading recognition tests, such as the REALM, are useful predictors of a person's reading ability. Although these tools do not directly assess comprehension, the assumption behind the reading recognition tests is that if a person cannot read a word aloud, it is unlikely for this person to have the ability to understand its meaning (Murphy et al., 1993). Moreover, because self-reported educational status is not a good predictor of reading ability, reading recognition tests are useful in clinic (Davis et al., 1993).

The REALM has standardized directions for administration and scoring. To participate, patients are asked to read a list of 66 common medical terms as well as lay terms for various health conditions and body parts aloud to the examiner. Target words increase in number of syllables and difficulty throughout the assessment. Participants are scored based on their ability to accurately pronounce the words. If a patient cannot read a word, they are instructed to make their best attempt or say “blank” and move on to the next item. Raw scores from the REALM can be used to determine grade range estimates for a person's reading abilities; however, results are “estimates of literacy, not grade equivalents” (Murphy et al., 1993, p. 126).

Although the original REALM had 125 words (Davis et al., 1991), the assessment was revised and shortened to increase its clinical utility to include 66 words (Davis et al., 1993). Bass et al. (2003) further shortened the 66-item REALM to only have 8 items (Rapid Estimate of Adult Literacy in Medicine-Revised [REALM-R]) as a rapid-screening tool. The results suggested that either the REALM or the REALM-R can be quickly administered, scored, and subsequently placed in a patient's chart to notify providers about a person's health literacy skills so they can adjust their communication accordingly.

Although domain-general health literacy tools such as the REALM have been developed for use in primary care settings, some medical specialties have also worked to develop domain-specific tools that assess an individual's health literacy skills regarding a specific topic, condition, or procedure. Examples of such tools include the Rapid Estimate of Adult Literacy in Dentistry (Richman et al., 2007), the Rapid Estimate of Adult Literacy in Vascular Surgery (Wallace et al., 2009), the Brief Estimate of Health Knowledge and Action—HIV Version (Osborn et al., 2010), and the Rapid Estimate of Adult Literacy in Genetics (Erby et al., 2008).

Currently, there are not any tools available to assess an individual's audiology-related health literacy. Hearing loss is a public health concern because the global population is aging at an unprecedented rate; therefore, the number of people with hearing loss will continue to increase in the next several decades (e.g., Lin et al., 2016). Although hearing loss is the third most common chronic physical condition in the United States, fewer than 25% of adults with hearing loss pursue intervention (e.g., Chien & Lin, 2012). Many patients and health care providers mistakenly believe that hearing loss is an inevitable part of aging (e.g., D'Haese et al., 2018; Walling et al., 2012). However, scientific studies have shown that hearing loss is independently associated with accelerated cognitive decline and a variety of other negative secondary physical and psychological health outcomes (e.g., Lin et al., 2013). There is an urgent need to increase accessibility and affordability of hearing health care (Donahue et al., 2010; Lin et al., 2016). Providing timely, evidence-based hearing health interventions for people with hearing loss is imperative. Research has indicated that low health literacy is a significant barrier to the use of existing effective health care services (Levy & Janke, 2016). A recent study used data from the Health and Retirement Study and found that the likelihood of using hearing aids was much lower for those with low self-assessed subjective health literacy (Munson Klyn et al., 2020). We suspected that people with limited audiology-related health literacy may not know how to access information and services, communicate their needs with providers, understand and make decisions about providers' recommendations, and identify information and services that align with their needs and priorities. Therefore, it is vital to assess health literacy specific to audiology before implementing interventions to improve access to hearing health care.

The purpose of the study was to develop an objective health literacy assessment tool in the audiology domain—the Rapid Estimate of Adult Literacy in Audiology (REALA).

## Materials and Methods

### Development of REALA

The REALA was adapted from the REALM and developed using the procedures outlined by Davis et al. (1991). The principal investigator of the study (H.O.) selected a total of 99 words or terms that were specific to the etiology, anatomy, diagnoses, and treatment options in the audiology field. All the chosen words were either commonly used in clinic or frequently presented in publicly available patient education materials, websites from organizations specializing in communication disorders, including the American Speech Language Hearing Association, the American Academy of Audiology, the Academy of Doctors of Audiology, and the National Institute on Deafness and Other Communication Disorders. A ten-member research team, which included one audiologist (H.O.), one speech patholo-gist and eight doctoral students of audiology, carefully reviewed all words for the preliminary version of the tool. The words were ranked in ascending order of difficulty based on the number of syllables and familiarity. On the stimulus form presented to participants, the 99 items were displayed in large, black font in four columns (**Table 1**) (May et al., 2020). Similar to the administration and scoring guidelines for the REALM, participants were instructed to read each item aloud and responses were marked as correct or incorrect. Responses were scored as *incorrect* if the target word was mispronounced, sounded out, skipped, or if a participant took more than five seconds to read the word aloud. The total score was the number of correct responses out of 99 words as a percentage.

**Table 1:**
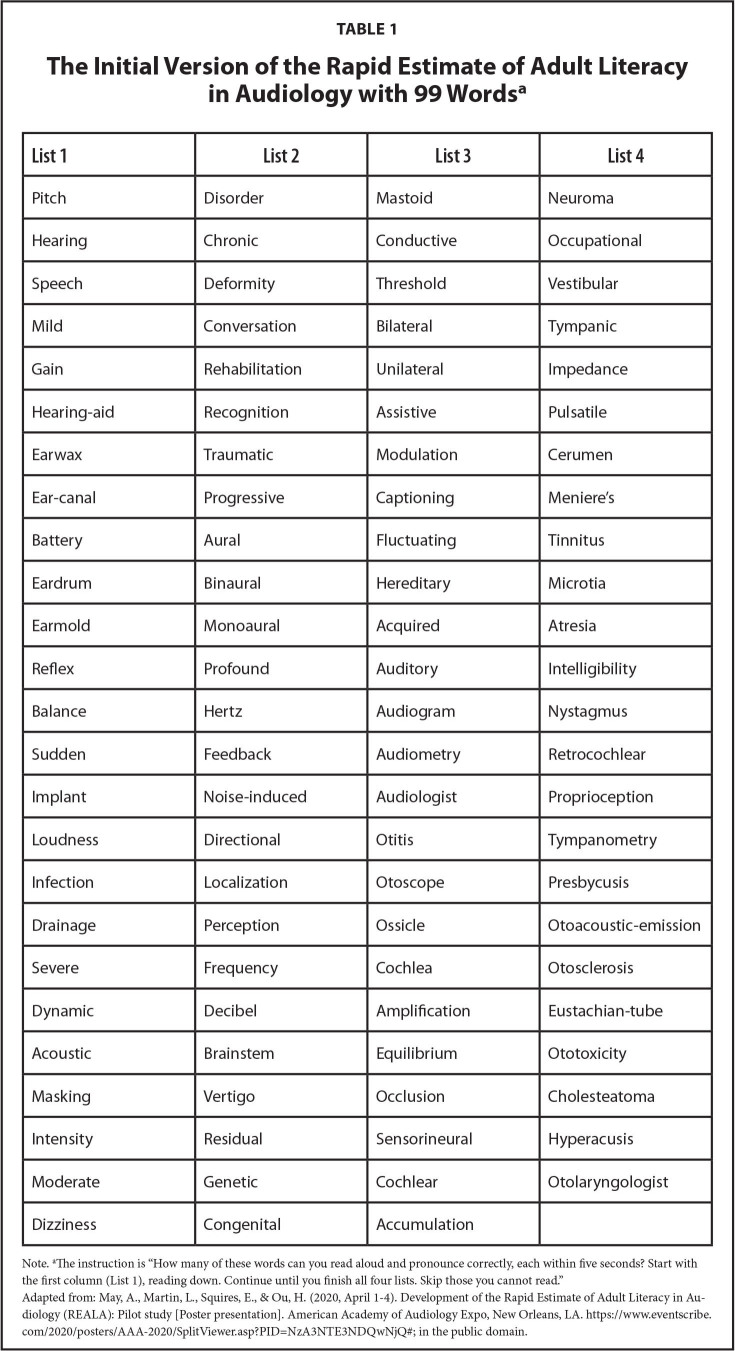
The Initial Version of the Rapid Estimate of Adult Literacy in Audiology with 99 Words^a^

**List 1**	**List 2**	**List 3**	**List 4**
Pitch	Disorder	Mastoid	Neuroma
Hearing	Chronic	Conductive	Occupational
Speech	Deformity	Threshold	Vestibular
Mild	Conversation	Bilateral	Tympanic
Gain	Rehabilitation	Unilateral	Impedance
Hearing-aid	Recognition	Assistive	Pulsatile
Earwax	Traumatic	Modulation	Cerumen
Ear-canal	Progressive	Captioning	Meniere's
Battery	Aural	Fluctuating	Tinnitus
Eardrum	Binaural	Hereditary	Microtia
Earmold	Monoaural	Acquired	Atresia
Reflex	Profound	Auditory	Intelligibility
Balance	Hertz	Audiogram	Nystagmus
Sudden	Feedback	Audiometry	Retrocochlear
Implant	Noise-induced	Audiologist	Proprioception
Loudness	Directional	Otitis	Tympanometry
Infection	Localization	Otoscope	Presbycusis
Drainage	Perception	Ossicle	Otoacoustic-emission
Severe	Frequency	Cochlea	Otosclerosis
Dynamic	Decibel	Amplification	Eustachian-tube
Acoustic	Brainstem	Equilibrium	Ototoxicity
Masking	Vertigo	Occlusion	Cholesteatoma
Intensity	Residual	Sensorineural	Hyperacusis
Moderate	Genetic	Cochlear	Otolaryngologist
Dizziness	Congenital	Accumulation	

Note.

a The instruction is “How many of these words can you read aloud and pronounce correctly, each within five seconds? Start with the first column (List 1), reading down. Continue until you finish all four lists. Skip those you cannot read.”

Adapted from: May, A., Martin, L., Squires, E., & Ou, H. (2020, April 1–4). Development of the Rapid Estimate of Adult Literacy in Audiology (REALA): Pilot study [Poster presentation]. American Academy of Audiology Expo, New Orleans, LA. https://www.eventscribe.com/2020/posters/AAA-2020/SplitViewer.asp?PID=NzA3NTE3NDQwNjQ#; in the public domain.

### Participants

We recruited 200 participants through fliers posted in local clinics and communities or word of mouth. All participants were adults, age 18 years or older, and the average participant age was 42.4 years (standard deviation [*SD*] = 17.6) with an age range from 18 to 84 years. Further demographic information, such as gender, race, and educational level as well as their hearing health history, is presented in **Table 2** (May et al., 2020). The participants were not paid for participating in the study.

**Table 2:**
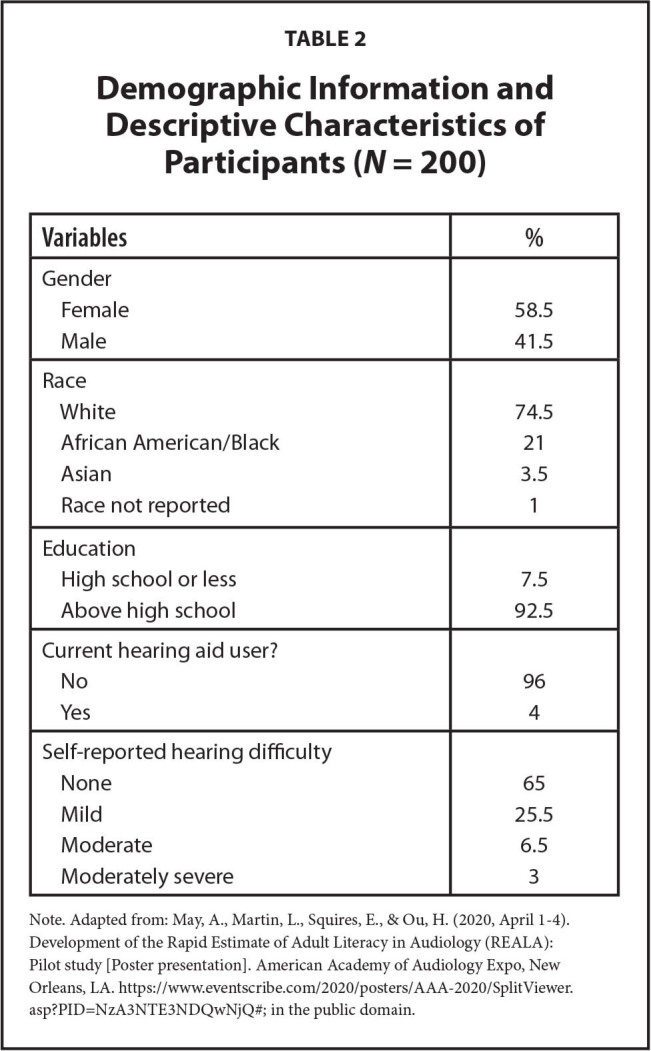
Demographic Information and Descriptive Characteristics of Participants (*N* = 200)

**Variables**	**%**
Gender	
Female	58.5
Male	41.5

Race	
White	74.5
African American/Black	21
Asian	3.5
Race not reported	1

Education	
High school or less	7.5
Above high school	92.5

Current hearing aid user?	
No	96
Yes	4

Self-reported hearing difficulty	
None	65
Mild	25.5
Moderate	6.5
Moderately severe	3

Note. Adapted from: May, A., Martin, L., Squires, E., & Ou, H. (2020, April 1–4). Development of the Rapid Estimate of Adult Literacy in Audiology (REALA): Pilot study [Poster presentation]. American Academy of Audiology Expo, New Orleans, LA. https://www.eventscribe.com/2020/posters/AAA-2020/SplitViewer.asp?PID=NzA3NTE3NDQwNjQ#; in the public domain.

### Procedure

In addition to the author, four graduate students were recruited to assist with data collection. All student research assistants were trained in how to administer the REALA and the REALM. All members of the research team collected preliminary data from five participants after an initial training session. The research team then met again to ensure that the tools were being administered and scored consistently and accurately. This study was deemed exempt from the Institutional Review Board at Wayne State University and participants did not need to sign a consent form; however, an information sheet that included the principal investigator's contact information was made available to all participants.

During the administration session, participants provided verbal consent and completed a form that requested demographic information. Next, participants were prompted to read the items on the preliminary version of the REALA aloud, skipping any items that they were unable to pronounce. Finally, the participants were presented with the REALM (Davis et al., 1993) and instructed to follow the same procedure as was used during completion of the REALA. We collected the data in a quiet room at the local community setting or at the principal investigator's laboratory.

### Data Analysis

Descriptive statistics are presented where appropriate. The classical item analysis was applied for the current study: (1) The item difficulty was based on the probability of pronouncing the item correctly from the total sample, which was described as the pass rate. The possible values of the pass rate ranged from 0 to 1; (2) The item discrimination refers to the ability of an individual test item to differentiate participants based on their knowledge of the material. The total percent correct score was first calculated across 99 words for each participant. Next, the participant was assigned to the *expert* group if the performance was above the 75th percentile of the data. If the participant performed below the 25th percentile, this person was assigned to the *novice* group. Otherwise, participants were excluded for the calculation of the item discrimination score. The average pass rate was further calculated for each item per group. Finally, the average pass rate of the *novice* group was subtracted from that of the *expert* group for each item to get the item discrimination score; (3) The point biserial index (PBI) was calculated for each item and it is the correlation between the score on a single item and the total score of the test. Indices below 0.2 were considered *poor*, indices between 0.2 and 0.29 were considered *fair*, indices between 0.3 and 0.39 were considered *good* and indices between 0.4 and 0.7 were considered *very good* (McGahee & Ball, 2009). Items with a higher discrimination score and PBI usually have a lower pass rate; therefore, providing the highest sensitivity when assessing an adult's hearing health literacy. Taken together, the pass rate, the discrimination score, and the PBI for each item was used to develop the final version of the tool that could be administered in a shorter amount of time, improving its clinical utility.

Given that the REALM has been correlated with other standardized measures, participants' scores on the REALM and the REALA were compared to test the convergent validity or construct validity of the REALA. The Cronbach coefficient alpha was used to assess the internal consistency of the items.

The data analyses were conducted through the Statistical Analysis System version 9.4^®^ software. For all tests, statistical significance was defined as *p* < .05.

## Results

### Classical Item Analysis for the Initial Version of REALA

The average percent correct score was 82.8% (*SD* = 12.1%) for the preliminary version of the REALA and 97.4% (*SD* = 6.5%) for the REALM in the current study across all participants. The pass rate for each item on the REALA ranged from 0.23 to 1 with the average pass rate of 0.83 (*SD* = 0.34). The 75th and 25th percentiles of the percent correct data from all 99 words for the REALA were 91.9% and 78.8% respectively. Based on the percentile information, those participants with a score higher than 91.9% were considered to have good performance (the *expert* group; *n* = 43) and those with a score lower than 78.8% were considered to have poor performance (the *novice* group; *n* = 65). The item discrimination score for each item was the difference between the pass rate of the *expert* and *novice* groups. The results indicated that item discrimination score ranged from 0 to 0.97 with the average value at 0.29 (*SD* = 0.30). The PBI was calculated accordingly for each item and the average PBI was 0.41 (*SD* = 0.17) and ranged from 0.02 to 0.62.

**Figure 1.**
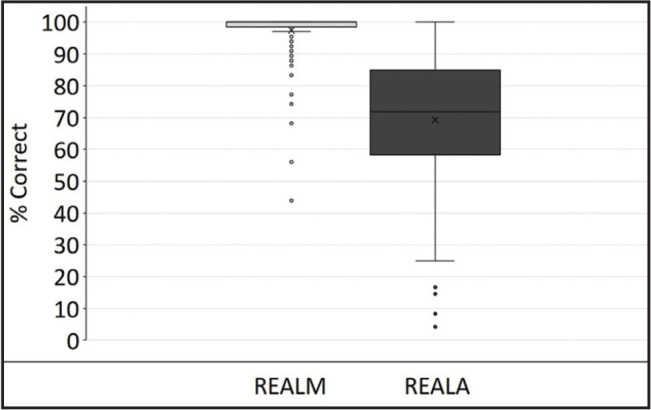
The Rapid Estimate of Adult Literacy in Audiology (REALA) and the Rapid Estimate of Adult Literacy in Medicine (REALM) total performance (% correct) across participants. The box represents the middle 50% of the data. The lower and upper outer lines that enclose the box represent the 25th and 75th percentiles, respectively. Solid horizontal lines in the box indicate the median. The whiskers represent the maximum and minimum values for each material. The circles represent the outliers of the individual data.

The initial version of REALA with 99 items was positively correlated with the REALM (*r* = 0.70, *p* < .0001). **Figure 1** displays the distribution of the raw data for both the REALA and the REALM (May et al., 2020).

### Development of the Final Version of REALA

The final version of REALA was developed to improve its clinical utility. It contained a total of 48 items that were selected based on the combination of item difficulty (i.e., pass rate), item discrimination score, and PBI using the classical item analysis. In general, PBI values higher than 0.30 are good. The primary criteria for the selection in the current study were based on the PBI. Those with values higher than 0.40 were chosen to stay. Then, among those chosen words, the items with a reasonable combination of low pass rate and high discrimination score were retained. **Table 3** displays the detailed information of item pass rate, item discrimination score, and PBI for the 48 selected words. The total pass rate for the final version of REALA was 0.72 (*SD* = 0.45).

**Table 3:**
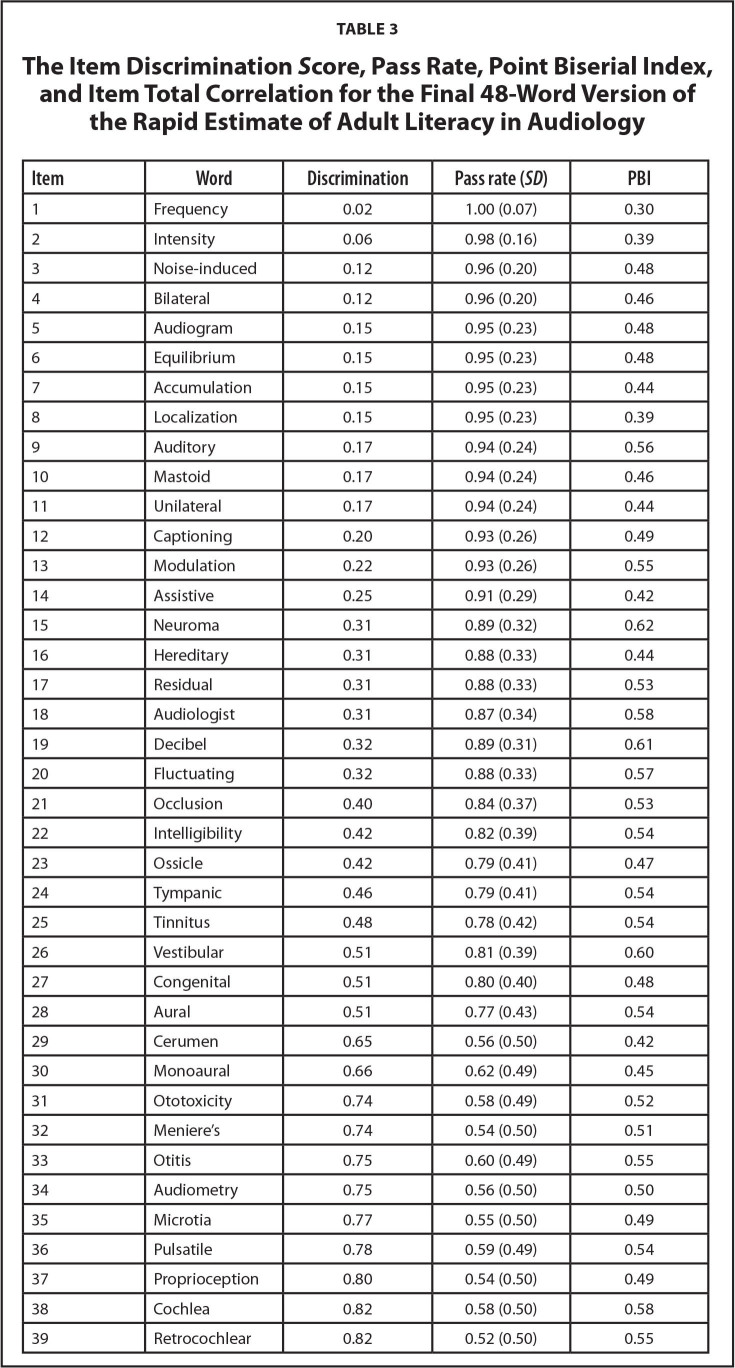

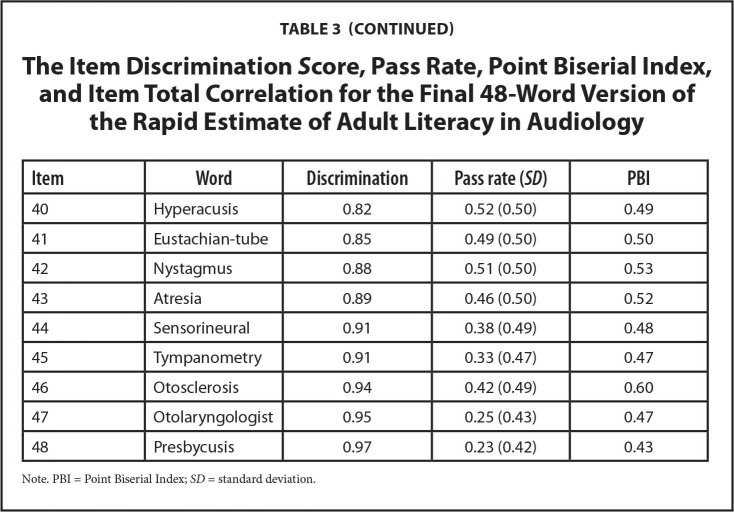
The Item Discrimination *S*core, Pass Rate, Point Biserial Index, and Item Total Correlation for the Final 48-Word Version of the Rapid Estimate of Adult Literacy in Audiology

**Item**	**Word**	**Discrimination**	**Pass rate (*SD*)**	**PBI**
1	Frequency	0.02	1.00 (0.07)	0.30
2	Intensity	0.06	0.98 (0.16)	0.39
3	Noise-induced	0.12	0.96 (0.20)	0.48
4	Bilateral	0.12	0.96 (0.20)	0.46
5	Audiogram	0.15	0.95 (0.23)	0.48
6	Equilibrium	0.15	0.95 (0.23)	0.48
7	Accumulation	0.15	0.95 (0.23)	0.44
8	Localization	0.15	0.95 (0.23)	0.39
9	Auditory	0.17	0.94 (0.24)	0.56
10	Mastoid	0.17	0.94 (0.24)	0.46
11	Unilateral	0.17	0.94 (0.24)	0.44
12	Captioning	0.20	0.93 (0.26)	0.49
13	Modulation	0.22	0.93 (0.26)	0.55
14	Assistive	0.25	0.91 (0.29)	0.42
15	Neuroma	0.31	0.89 (0.32)	0.62
16	Hereditary	0.31	0.88 (0.33)	0.44
17	Residual	0.31	0.88 (0.33)	0.53
18	Audiologist	0.31	0.87 (0.34)	0.58
19	Decibel	0.32	0.89 (0.31)	0.61
20	Fluctuating	0.32	0.88 (0.33)	0.57
21	Occlusion	0.40	0.84 (0.37)	0.53
22	Intelligibility	0.42	0.82 (0.39)	0.54
23	Ossicle	0.42	0.79 (0.41)	0.47
24	Tympanic	0.46	0.79 (0.41)	0.54
25	Tinnitus	0.48	0.78 (0.42)	0.54
26	Vestibular	0.51	0.81 (0.39)	0.60
27	Congenital	0.51	0.80 (0.40)	0.48
28	Aural	0.51	0.77 (0.43)	0.54
29	Cerumen	0.65	0.56 (0.50)	0.42
30	Monoaural	0.66	0.62 (0.49)	0.45
31	Ototoxicity	0.74	0.58 (0.49)	0.52
32	Meniere's	0.74	0.54 (0.50)	0.51
33	Otitis	0.75	0.60 (0.49)	0.55
34	Audiometry	0.75	0.56 (0.50)	0.50
35	Microtia	0.77	0.55 (0.50)	0.49
36	Pulsatile	0.78	0.59 (0.49)	0.54
37	Proprioception	0.80	0.54 (0.50)	0.49
38	Cochlea	0.82	0.58 (0.50)	0.58
39	Retrocochlear	0.82	0.52 (0.50)	0.55
40	Hyperacusis	0.82	0.52 (0.50)	0.49
41	Eustachian-tube	0.85	0.49 (0.50)	0.50
42	Nystagmus	0.88	0.51 (0.50)	0.53
43	Atresia	0.89	0.46 (0.50)	0.52
44	Sensorineural	0.91	0.38 (0.49)	0.48
45	Tympanometry	0.91	0.33 (0.47)	0.47
46	Otosclerosis	0.94	0.42 (0.49)	0.60
47	Otolaryngologist	0.95	0.25 (0.43)	0.47
48	Presbycusis	0.97	0.23 (0.42)	0.43

Note. PBI = Point Biserial Index; *SD* = standard deviation.

**Table 4:**
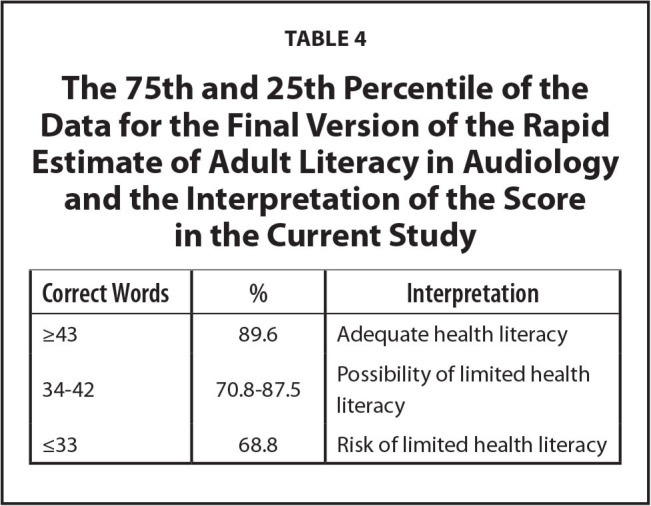
The 75th and 25th Percentile of the Data for the Final Version of the Rapid Estimate of Adult Literacy in Audiology and the Interpretation of the Score in the Current Study

**Correct Words**	**%**	**Interpretation**
≥43	89.6	Adequate health literacy
34–42	70.8–87.5	Possibility of limited health literacy
≤33	68.8	Risk of limited health literacy

It should be noted that the first two items with high pass rates listed in **Table 3** were retained based on an approach used by Bass et al. (2003) who developed the REALM-R. The intention was that beginning a reading recognition assessment with the items that have a high pass rate could help decrease test anxiety and improve confidence.

The Cronbach's alpha coefficient was 0.93 for the final version of REALA. The results indicated high internal consistency between the items. That is, the words were likely measure the same construct.

**Table 4** displays the 75th and 25th percentiles of the data for the final version of the REALA across participants in the current study. Individuals who received a score greater than or equal to 43 out of 48 items correct performed at or above the 75th percentile and can be classified as having sufficient audiology-related health literacy. Alternatively, individuals who received a score of 33 or lower performed at or below the 25th percentile of the participants and can be classified as having risk of limited audiology-related health literacy. It should be noted that the normative data to be used to rate the audiology-related health literacy level should be developed separately for different target population.

## Discussion

Health literacy is an under-studied area in hearing health care and there are no tools available to specifically assess audiology-related health literacy. The purpose of this study was to develop a health literacy assessment tool specific to audiology for both clinical and research settings. Results suggested that the final 48-item version of the REALA appears to be an encouraging tool of hearing-health specific health literacy based on its strong psychometric strengths, significant correlation with a widely used general health literacy assessment tool and its good internal reliability.

The REALA can help professionals to avoid using field-specific jargon with patients by identifying the audiology-related terms that are most difficult for people to understand. For example, the top five missed words in the study were “presbycusis,” “otolaryngologist,” “cholesteatoma,” “tympanometry,” and “sensorineural,” which suggests that the average person may not be familiar with these terms. Therefore, a provider could briefly examine their patient's responses on the REALA prior to an appointment and provide more common vocabulary such as “age-related hearing loss” rather than “presbycusis” or explain that presbycusis is a type of hearing loss commonly associated with aging. Another term included in the REALA that is commonly used by providers in clinical practice is the term “tinnitus,” which only had 78% pass rate. Given the fact that 92.5% of participants reported having education beyond a high-school degree, the findings from this study indicated that health professionals cannot make assumptions about a client's ability to understand those words via oral or written communication based on their educational history. It is suggested that field-specific jargon should be replaced with plain English whenever possible.

Furthermore, although the REALA scores were significantly correlated with the REALM scores, **Figure 1** showed that participants in general performed worse for the REALA compared to the REALM. This finding indicated that it is critical to assess domain-specific health literacy (May et al., 2020).

In addition, it is interesting to note that the average REALA score was comparable between those who self-reported hearing difficulty (mean = 83.68%; *SD* = 13.07%; *n* = 70) and those who reported no hearing difficulty (mean = 82.39%; *SD* = 11.62%; *n* = 130). The results indicated that the level of audiology-related health literacy was not impacted by their status of hearing from the current study.

Once the comprehension component is added to the tool, implementing the REALA into routine clinical practice can allow hearing health professionals, such as primary care physicians, ear, nose, and throat doctors, Audiologists, and hearing aid specialists, to quickly administer and score this tool in-office. In return, the information can help professionals tailor education and intervention plans based on clients' health literacy needs.

Additionally, we need to be cautious about patients with dysphonia. There is a relatively high co-prevalence (∼10%) of hearing loss and dysphonia among older adults (e.g., Cohen & Turley, 2009). Because the REALA scoring is based on pronunciation of those words, hearing health professionals should be aware of the issue of dysphonia and take it into account for test and re-test as well as the needed training for scoring.

## Study Limitations

It should be noted that the tool used in this study to assess audiology-related health literacy was developed based on the assumption that if a person cannot read a word correctly, the likelihood that they would be able to understand the meaning of this word is low. Therefore, the tool did not directly assess a person's ability to comprehend word meaning. However, a study about dental health literacy assessment demonstrated a significantly strong correlation between recognition and comprehension measures (Khan et al., 2014). In Khan et al. (2014) study, it appeared that participants' reading recognition scores were typically higher than their comprehension scores, which suggested that when reading recognition tests were used as an indirect measure of comprehension, a person's comprehension skills may be overestimated. Therefore, it is a good idea to use the REALA conservatively.

Second, the sampling of the study was based on the convenience sampling; therefore, generalization is limited and the results from this study cannot represent health literacy at the population level.

Lastly, the test-retest reliability of the final version was not rigorously assessed. The future direction of this research is to rigorously evaluate the test-retest reliability and to add a comprehension component to the REALA.
